# Ultra-Sensitive Bioanalytical Separations Using a New 4-Tritylphenyl Methacrylate-Based Monolithic Nano-Column with an Inner Diameter of 20 µm for Nano-LC

**DOI:** 10.3390/ijms27010224

**Published:** 2025-12-25

**Authors:** Cemil Aydoğan

**Affiliations:** 1 Food Analysis and Research Laboratory, Bingöl University, Bingöl 12000, Türkiye; caydogan@bingol.edu.tr; Fax: +90-426-216-00-33; 2 Department of Food Engineering, Bingöl University, Bingöl 12000, Türkiye; 3 Department of Chemistry, Bingöl University, Bingöl 12000, Türkiye

**Keywords:** miniaturization, monolith, nano-liquid chromatography, peptide, protein, proteomics, ultralow flow LC

## Abstract

Low-flow liquid chromatography has become the primary tool for advanced chromatographic analysis and is an indispensable technique for the sensitive detection of biomolecules. In this study, we developed a new 4-tritylphenyl methacrylate-based monolithic nano-column with an internal diameter of 20 µm for bioanalytical separations in nano-liquid chromatography (nano-LC). The composition of the monolith was optimized with regard to the monomer and porogenic solvent. The column was characterized using Fourier Transformed Infrared Spectroscopy (FT-IR) spectroscopy, scanning electron microscopy (SEM) and chromatographic analyses. Chromatographic characterization was performed using homologous alkylbenzenes (ABs) and polyaromatic hydrocarbons (PAHs), which facilitate hydrophobic and π–π interactions. Run-to-run and column-to-column reproducibility values were found to be <2.51% and 2.4–3.2%, respectively. The final monolith was then used to separate six standard proteins, including β-lactoglobulin A, carbonic anhydrase, ribonuclease A (RNase A), α-chymotrypsinogen (α-chym), lysozyme (Lys), cytochrome C (Cyt C) and myoglobin (Myo), as well as three dipeptides: Alanine-tyrosine (Ala-Tyr), Glycine-phenylalanine (Gly-Phe) and L-carnosine. The nano-column was then applied to profiling peptides and proteins in the MCF-7 cell line, enabling high-resolution peptide analysis.

## 1. Introduction

Low-flow liquid chromatography has become the primary tool for advanced chromatographic techniques and is indispensable for the sensitive separation of biomolecules [1]. A notable trend in modern chromatography is the increasing use of miniaturized columns for rapidly and sensitively analyzing small-volume, complex samples. Narrow bore columns (e.g., ≤100 µm ID) with flow rates of up to 1000 nL/min are widely used, enabling greater sensitivity. Among other low flow rate LC techniques, nano-LC is an advanced chromatographic technique used for proteomics [2] metabolomics [3], lipidomics [4] and foodomics [5], due to its ability to analyze small-volume samples with high sensitivity, reducing radial dilution and achieving high efficiencies. These separations are used to determine low-abundant compounds in limited samples [2]. A key factor in the dilution process is the use of a narrow analytical column. These include micro-scale columns with an ID of 1 mm and capillary scale columns with an ID of 0.2–0.5 mm. Narrow bore columns (e.g., ≤100 µm ID) with flow rates of up to 1000 nL/min are widely used in nano-LC, enabling greater sensitivity. There are several types of narrow column, including monolithic, packed, open-tubular and chip-format columns [6]. These miniaturized columns are increasingly being used for the rapid and sensitive analysis of small, complex samples [7]. Among these, monolithic columns are a promising alternative for low flow rate LC separations. Although packed columns are mostly used for nano-LC, monolithic columns offer several advantages, such as ease of preparation and a large surface area [8]. The hydrodynamic properties of monolithic and packed columns differ in several ways. For example, diffusion and pore size are major limitations of packed columns, which are only utilized to a limited extent, whereas convection is the main factor in monoliths, facilitating interphase mass transfer [9]. Another format is the open-tubular column, which is characterized by a lower amount of stationary phase on the column [10]. In contrast, pillar array columns are a new technology that provides high separation performance at lower pressure. However, these columns are very expensive [11]. The chip format offers limited flexibility. Therefore, developing new monolithic columns for nano-LC is crucial for modern chromatography.

Monolithic columns are widely used in the separation of proteins and peptides [12,13,14]. Proteomics analysis of extremely limited samples is crucial and requires advanced analytical tools due to the dual challenges presented by proteomics applications. Firstly, analyzing proteins at very low concentrations is difficult, and secondly, the volume of the proteome sample results in an extremely low volume. In a previous study, we developed a poly-L-Lysine-based open-tubular column with an inner diameter of 20 µm that features both electrostatic and hydrophobic interactions for separating casein protein variants [15]. By using pL/min–nL/min flow rates, nano-LC-based columns can enable the sensitive detection of low-abundant biomolecules in samples, providing striking results.

This study presents the development and application of a new monolithic nano-column based on 4-tritylphenyl methacrylate for sensitive bioanalytical separations in nano-LC. Since the monolith incorporated the advantages of 4-TPM, the influence of various parameters was investigated, including the concentration of 4-TPM in the polymerization mixture. The final 4-TPM monolith was then used to separate six standard proteins and three peptides. It was also used to analyze the MCF-7 cell line via nano-LC with linear gradient separation.

## 2. Results and Discussion

### 2.1. The Preparation and Characterization of 4-TPM-Based Monolithic Column

As the H and C NMR results show, the presence of numerous proton signals in the 7.0–7.3 ppm range in the spectrum confirms the presence of aromatic rings (–CH_2_). The signals at 6.25 and 5.88 ppm correspond to vinylic protons (C=CH_2_), while the signal at 1.98 ppm corresponds to a methyl group (CH_3_). This distribution indicates the presence of both aromatic structures and vinylic functionalities. Regarding the carbon nuclear magnetic resonance (C NMR) spectrum, the signal at 18.67 ppm indicates methyl carbon, the signal at 64.79 ppm indicates vinyl carbon, the signals in the 121–149 ppm range indicate aromatic carbons and the signal at 165.83 ppm likely indicates a carbon belonging to a conjugated carbonyl/heteroaromatic structure. In conclusion, the spectra confirm the presence of the expected aromatic rings, vinylic groups and methyl group in the 4-TPM monomer structure.

Optimizing the content of the monomer and porogen, as well as other parameters such as time and temperature, is crucial in preparing new monoliths. These parameters are key to achieving efficient chromatographic separation. 4-TPM was synthesized and used as the main monomer in the polymerization mixture due to its hydrophobic structure, which is important for proteomics analysis. Although ethylene dimethacrylate (EDMA) is mostly used as a cross-linker for methacrylate monoliths, TRIM (a tri-functional monomer) was chosen as the cross-linker, as this may enable the formation of macroporous polymers under relevant conditions [16]. Using TRIM may contribute to the efficient transport of hydrophobic macromolecules through flow-through pores and provide good loading capacity. Regarding the porogenic solvent, we experimented with several systems, including toluene:1-dodecanol, THF:1-dodecanol, propanol:1-dodecanol and cyclohexanol:1-dodecanol. The 4-TPM showed good solubility in these porogenic solvents. However, the hydrodynamic properties and robustness of the prepared monoliths, except for those prepared using cyclohexanol:1-dodecanol, were not satisfactory for forming the desired porous structure, which may be due to the solubility properties (δ) of the porogenic solvent [17]. Table 1 shows the composition of the polymerization mixtures used to prepare the 4-TPM monoliths. The 4-TPM content of the polymerization mixture was investigated while keeping the TRIM content constant, which is crucial for preparing a highly hydrophobic monolith. The 4-TPM content was varied from 0.88% to 21.17% (*w*/*w*). The optimized 4-TPM content was found to be 18.21% (*w*/*w*) in the final polymerization solution and further increasing the 4-TPM monomer content resulted in the formation of a monolith with no flow. As shown in Table 1, the surface area of the optimized 4-TPM monolith (e.g., TPM 6) was calculated to be 211.7 m^2^/g. The specific surface area increased with the increasing content of 4-TPM, and the optimized molar ratio of 4-TPM to TRIM was found to be 1.2:2.1 (*v*/*v*). The developed monoliths were characterized by FT-IR spectroscopy. Specific bands stemming from the structure of 4-TPM were determined in the spectra, including aromatic C-H stretching at around 3080 cm^−1^ (see Figure S5). Aromatic C-H bending at 750.82 cm^−1^ could be assigned to the vibrations of the 4-TPM-incorporated monolith. The increasing content of 4-TPM in the monoliths could also be seen in the FT-IR spectra.

**Figure 1:**
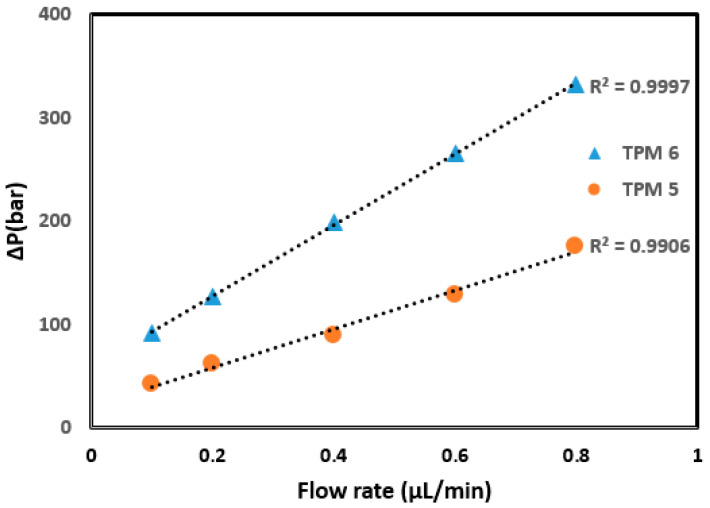
Linear relationship between flow rate and backpressure for the columns TPM 5 and 6.

Some of the hydrodynamic properties of monolithic columns containing different amounts of 4-TPM are given in Table 1. As can be seen, permeability could be achieved using the porogenic solvent system of cyclohexanol and 1-dodecanol in a 1:4 ratio. The TPM 5 monolith permitted permeability when ACN was used as the mobile phase, and the TPM 6 monolith exhibited improved permeability. The monoliths exhibited insufficient flow when the 4-TPM content was increased (TPM 7, 8). This could be explained by the increased 4-TPM content causing the formation of microglobules and thus leading to high backpressure. The backpressure value for the TPM 6 monolith showed a good linear relationship (R^2^ = 0.9997) between flow rate and resulting backpressure, whereas a weaker relationship (R^2^ = 0.9906) was obtained for the TPM 5 column (see Figure 1). The column also demonstrated a stable performance over several hundred injections under typical operating conditions before any noticeable degradation occurred. These results indicate that TPM content affects column stability, suggesting that the TPM 6 monolith has good mechanical stability.

Figure S4 shows the linear velocity on the plate height of the TPM 6 monolith using ACN H_2_O (80/20% (*v*/*v*)) for the separation of alkylbenzene derivatives. The monolitihc column efficiencies could reach up to 32,700–46,400 plates/m at optimum flow velocity. This result showed that the developed monolith may provide a more rapid mass transfer. The nitrogen physisorption was used for the determination of the specific surface area for the TPM 5 and TPM 6 monolithic columns, which was calculated by the Brunauer–Emmett–Teller method. The surface area for the columns the TPM 5 and the TPM 6 was calculated as 141.4 and 211.7 m^2^/g, respectively (see Table 1). This result is convincing proof for using TRIM as a crosslinker, as it can be used for the monolith preparation of 4-TPM.

**Figure 2:**
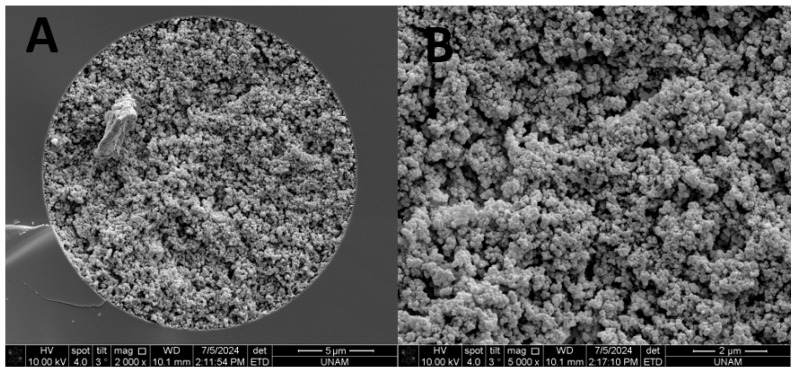
SEM images of the TPM 6 column with mag 2000× (**A**) and with 5000× (**B**).

The SEM images for the TPM 6 monolith were given in Figure 2. As shown here, morphology of the TPM 6 column showed a homogenous structure while a good attachment to the inner wall of capillary could be obtained.

The robustness of the developed TPM 6 monolith was evaluated in terms of percent relative standard deviations (RSDs) of *k* values of the test solutes including both uracil (*k*_av_ = 0.54) and toluene (*k*_av_ = 5.81). RSD % values were found to be less than 1.6%, indicating the robustness of the TPM 6 column. Five TPM 6 monolithic columns were prepared under the same polymerization conditions in order to evaluate the column-to-column reproducibility. With a total of 30 different runs, RSD values % were found as 2.64 and 1.27 for uracil and toluene, respectively. The RSDs of column-to-column, run-to-run and batch-to-batch tests were less than 2.7, 2.5 and 3.1% (*n*  =  5), respectively. The RSD of retention time was less than 3% for all analyses tried. These results showed the developed monolith could be prepared and used with satisfactory reproducibility. The developed TPM 6 monolith yielded an average of 9 µm for uracil, which produce over N ≈ 46.400 plates/m. These results indicated that TPM 6 monolith could use a wide range of low flow rates.

### 2.2. Chromatographic Evaluation

**Figure 3:**
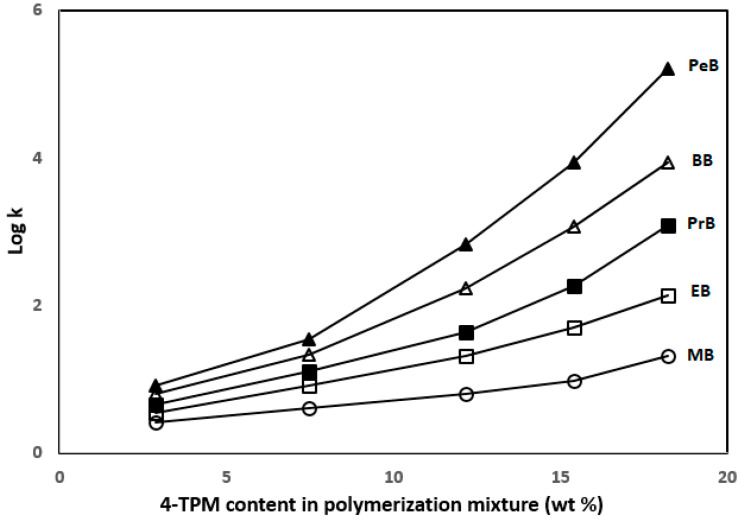
Plot of log *k* of ABs versus 4-TPM content in the polymerization mixture.

Chromatographic characterization of the TPM 6 column was performed using ABs, including methylbenzene (MB), ethylbenzene (EB), propylbenzene (PB), butylbenzene (BB) and pentylbenzene (PB) with different ACN content in the mobile phases at various flow rates. Figure 3 shows log *k* values versus 4-TPM content. It can be seen from here, the retention of ABs increased with increasing 4-TPM content in the columns.

**Figure 4:**
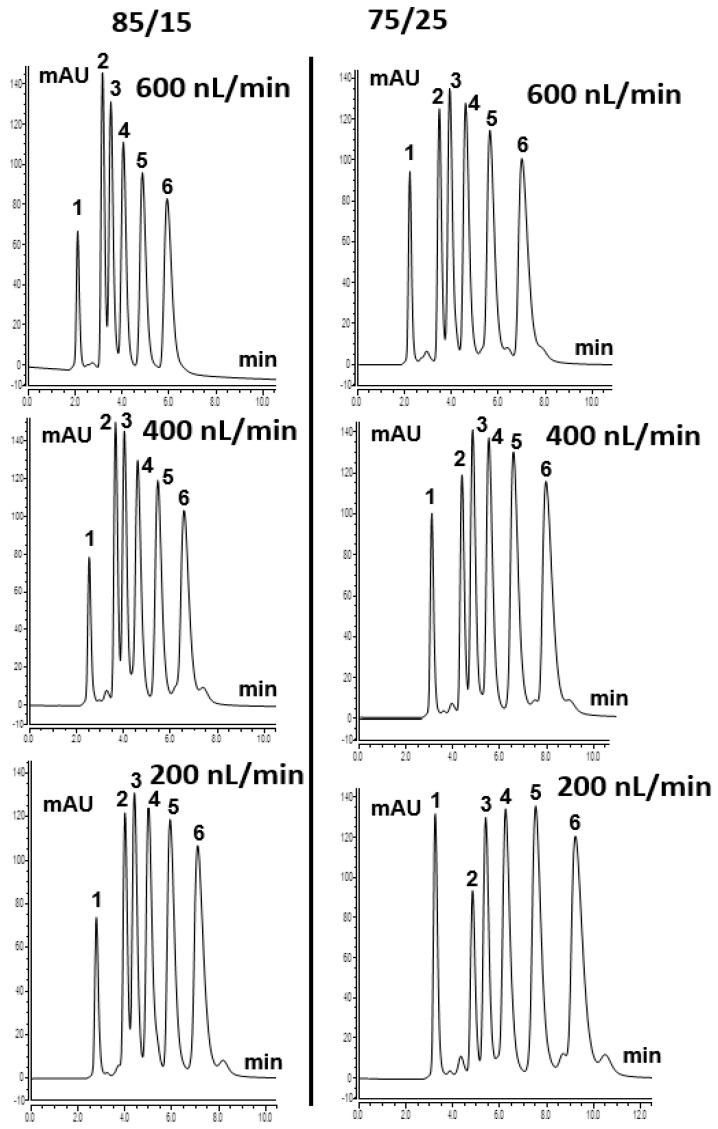
ABs separation chromatogram with the mobile phase (85/15% and 75/25% ACN/H_2_O (*v*/*v*)) using TPM 6 monolith at various flow rates. Order of peaks: (1) uracil; (2) methylbenzene (MB); (3) ethylbenzene (EB); (4) propylbenzene (PrB); (5) butylbenzene (BB); (6) pentylbenzene (PeB).

ABs separation can be attributed to the logarithmic methylene group selectivity (log_αCH2_) [18]. TPM 6 column was also tested using various ACN content from 75% to 85% in the mobile phase. Homologous ABs were well separated on the column at a higher content of ACN, which shows the typical RP mechanism. Figure 4 shows the separation chromatograms of homologous ABs with different ACN content at various flow rates. At the more aqueous 75/25 ACN/H_2_O (*v*/*v*) conditions, decreasing the flow rate from 400 to 200 nL/min increases the residence time of the mobile phase within the highly hydrophobic 4-TPM monolith. For the weakly retained polar compound uracil, this longer residence time slightly increases its dispersion and apparent breakthrough volume. Consequently, a larger proportion of uracil elutes within the acquisition window at 200 nL/min. In contrast, methylbenzene interacts more strongly with the hydrophobic stationary phase. At the lower flow rate, its retention and partial on-column focusing are enhanced. This results in a lower eluted amount under identical gradient and detection conditions. Overall, the results showed that the developed TPM 6 column could be used to separate hydrophobic compounds and that the column indicated an RP separation mechanism originating from 4-TPM, as described in the literature [19].

**Figure 5:**
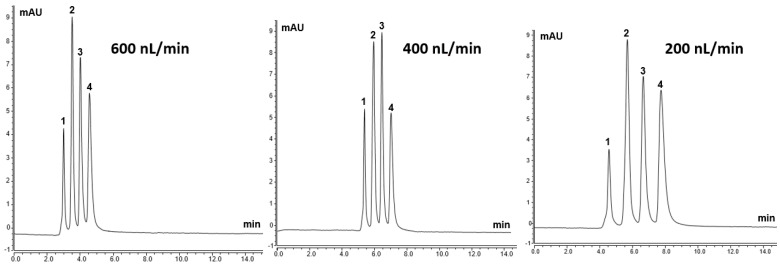
The separation chromatograms of PAHs using TPM 6 column at different flow rates with the mobile phase of ACN:H_2_O (86:14%, *v*/*v*). Peak order: 1—napthalene, 2—antracene, 3—phenantherene and 4—pyrene.

In order to test electron donating ability, which is crucial in the separation of peptides and proteins, polyaromatic structures, such as the surface phenyl ligands, were considered for the column chromatographic characterization. In this sense, TPM structures are known as efficient donors of π-electrons. Therefore, the TPM 6 monolith was considered to have electron donating ability towards polyaromatic hydrocarbons (PAHs), which have electron accepting ability. Further PAHs testing of the TPM 6 column was carried out, as 4-TPM may include both a strong π-π stacking interaction and an electron donating effect towards the aromatic solutes. Four different PAHs, including napthalene, antracene, phenantherene and pyrene, were separated on the TPM 6 column at different flow rates as the column may include both an electron donating effect and a strong π-π stacking interaction towards the aromatic solutes [20]. Figure 5 shows the separation chromatograms of PAHs using the TPM 6 column at different flow rates (e.g., 600 nL/min, 400 nL/min and 200 nL/min). Using the mobile phase of ACN:H_2_O (86:14%, *v*/*v*) the good separation chromatograms of PAHs could be achieved. The apparent differences in the relative areas of PAH peaks between 200 and 400 nL/min are due to experimental factors rather than genuine changes in elution stoichiometry. These factors include: (i) small variations in injection volume and detector response at ultra-low flow; (ii) slight differences in extra-column dispersion and baseline noise; and (iii) the use of different integration windows for partially overlapping peaks at the two flow rates. The PAH mixture concentration and composition were identical, and no selective loss or discrimination on the column was observed. The results showed that the column interacted well with PAHs, which showed that the column had a good electron donating ability under RP chromatography conditions.

### 2.3. Peptide and Protein Separation

The separation of peptides was investigated using the monolithic nano-column at various mobile phase pH levels. Three dipeptides, Ala-Tyr, Gly-Phe and L-carnosine were separated using nano-LC with the developed TPM 6 column. The best separation was achieved at neutral pH levels (e.g., pH 7.0). Figure S1 shows the chromatogram of the separation of the three dipeptides at different pH values. As the pH increased, significant peak broadening was observed for the tested peptides. In this separation, the nano-column exhibited strong hydrophobic retention and π-π interactions. Same chromatographic conditions were applied for the separation of the peptides [8]. As the pH increased, significant peak broadening was observed for the tested peptides. This was due to the charged groups on the peptides, which were not interacting with the monolithic structure. The best separation was achieved at pH 7.4. Figure S6 shows the chromatogram of the separation of the three dipeptides. The separation mechanism included strong π-π and hydrophobic interactions.

**Figure 6:**
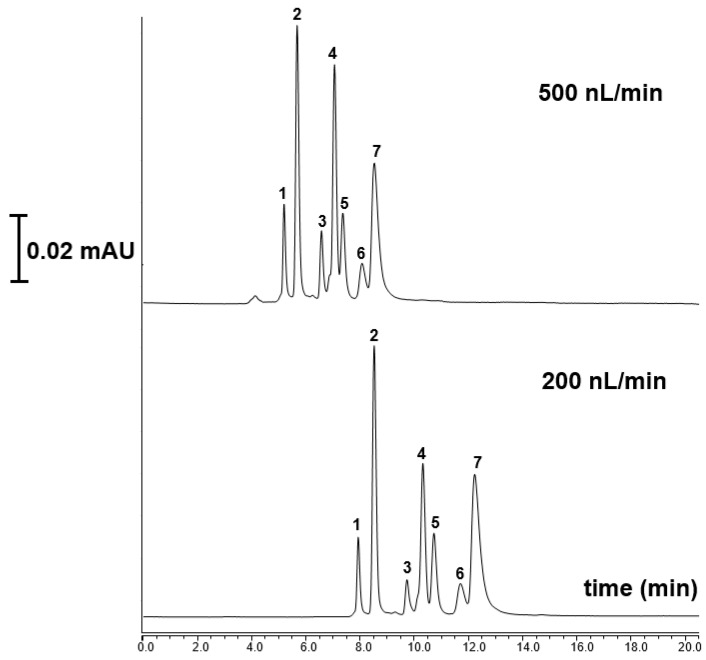
The separation chromatograms of standard proteins using the TPM 6 column at different flow rates chromatographic conditions: mobile phase A: 10% ACN/90% H_2_O at 0.1% *v*/*v* TFA; mobile phase B: 90% ACN/10% H_2_O at 0.1% *v*/*v* TFA; Peak order: 1—RNase A, 2—cytochrome C, 3—carbonic anhydrase, 4—Lys, 5—Myoglobin, 6—β-lactoglobulin A and 7—α-Chym A.

Nano-LC based protein separation is a rapidly advancing technique that aims to dramatically reduce the time required to isolate and analyze proteins, which is crucial for applications in proteomics analysis. Proteomics analysis employs nano-LC with ultra-narrow bore columns and nL/min flow rates [21,22]. In this study, seven standard proteins, including α-Chymotryp A (pI 8.75) cytochrome C (pI 10.0–10.5), carbonic anhydrase (pI, 6.9–7.0) Lys (pI 11.0), Myoglobin (pI 6.88–7.33), β-lactoglobulin A (pI = 5.1) and RNase A (pI 9.6) were readily separated using the TPM 6 monolith under linear gradient elution conditions. In this separation, mobile phase A was 10% ACN/90% H_2_O at 0.1% *v*/*v* TFA while mobile phase B 90% ACN/10% H_2_O at 0.1% *v*/*v* TFA which was applied according to a previous study [8]. Figure 6 shows the separation chromatograms of seven standard proteins with different flow rates. As shown here, the developed column showed a high resolving separation of the proteins, while lowering the flow rate from 500 to 200 nL/min allowed an increasing of retention time of proteins.

The results showed that the separation mechanism included a hydrophobic and π-π interactions column between the column surface hydrophobicity and amino acid residue for protein separation [23,24]. With the developed TPM 6 column, the separation of six standard proteins could be successfully achieved in nano-LC while it was shown that the column demonstrated a good capability protein separation, indicating a promising material for top-down proteomics analysis.

### 2.4. Proteomics Analysis

**Figure 7:**
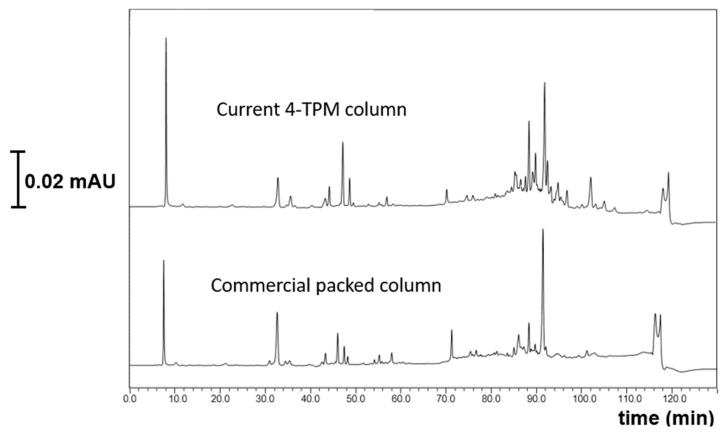
Proteomics analysis of MCF-7 cell line with diluted conditions at gradient elution 5–42% B, 200 nL/min 20 cm column lengths detection wavelength 214 nm.

Trace proteomics analysis refers to a cutting-edge approach in proteomics that increases the speed and throughput of protein analysis using ultra-narrow bore columns, which allows the identification of more than 5000 proteins in a single operation when coupled with high resolution mass spectrometers [25]. There are two different proteomics analysis approaches including, protein centric (top down) and peptide centric (bottom-up) [26]. The most common one is the use of the peptide centric approach. In this sense, we tested the proteomics analysis of the MCF-7 cell line using both the current developed monolith (e.g., TPM 6) and a commercial column. In preliminary optimization, the commercial 20 µm i.d. column was evaluated over a range of nanoflow rates (200–500 nL/min) under the same gradient profile. Its best compromise between peak capacity, backpressure and robustness was obtained around 400 nL/min, which is why this condition was selected for the comparison. Nano-LC conditions were applied under gradient elution, such as 2–35%B at a flow rate of 400 nL/min, at 17 cm in length and applying a gradient time of 120 min. The high resolving power of TPM 6 monolith was applied for the proteomics analysis of the MCF-7 cell line in the nano-LC system. The developed TPM 6 monolith had high resolving power for proteomics analysis, allowing for a high peak capacity as well as for the sensitive separation of peptides. When comparing with the commercial counterpart with 20 µm i.d., more sensitive and selective results with respect to both peptide resolution and better separation performance could be achieved (see Figure 7). The results proved that the developed column could be a promising alternative for trace proteomics analysis.

## 3. Materials and Methods

### 3.1. Chemicals and Reagents

4-Tritylphenyl methacrylate (4-TPM) was synthesized in our laboratory (see the next section for details). Trimethylolpropane trimethacrylate (TRIM) was purchased from Sigma-Aldrich (St. Louis, MO, USA). Homologous alkylbenzenes including methylbenzene (MB), ethylbenzene (EB), propylbenzene (PB), butylbenzene (BB), pentylbenzene (PB) as well as the polyaromatic hydrocarbons (PAHs) including napthalene, anthracene, phenantherene and pyrene were purchased from Merck A.G (Darmstadt, Germany). Fused silica capillary column with a 20 µm ID were purchased from BGB Analytik (Istanbul, Türkiye). Two quick-mount internal unions (JR-C360QUPK4) with a diameter of 360 µm from ViciValco (CH-8484 Weisslingen, Switzerland) were used for the connection between the trap column and the developed analytical monolithic column. L-carnosine, Ala-Tyr and Gly-Phe were purchased from Sigma Aldrich (St. Louis, MO, USA). Seven standard proteins, including RNase A, Lys, Cyt C, Mb, β-lactoglobulin, carbonic anhydrase and α-Chymotryp A were purchased from Sigma Aldrich (St. Louis, MO, USA).

### 3.2. Instrumentation

The NCS-3500RS Nano ProFlow (5041.0010A) manufactured by Dionex (Thermo Scientific, Waltham, MA, USA) was used for nano-LC experiments. This system includes the following components: a VWD-3400RS detector with a 3 nL flow cell and a WPS-3000TPL RS. Ultrapure water (18 MΩ cm resistivity) required for use in the analysis was produced using a purification system from Millipore Corporation (Billerica, MA, USA). Scanning electron microscopy (SEM; Zeiss, Evo-50, Jena, Germany) was used to examine the surface morphology of the columns. Ultrapure water was obtained using a Direct-Q^R^-3 from Millipore corporation (Billerica, MA, USA). The specific surface area was calculated using the BET equation via the Nov. 2200 E (Malvern Instruments Ltd., Worcestershire, UK), based on the nitrogen adsorption–desorption isotherm. Thin-layer chromatography (TLC) was used to monitor the reaction process and 4-TPM monomer synthesis was monitored using precoated silica plates with a thickness of 0.25 mm. A Bruker 400 (100)-MHz spectrometer (Ettlingen, Germany) was used to obtain ^1^H and ^13^C NMR spectra.

### 3.3. The Preparation of the 4-TPM-Based Monolithic Column

#### 3.3.1. Monomer Synthesis

**Figure 8:**
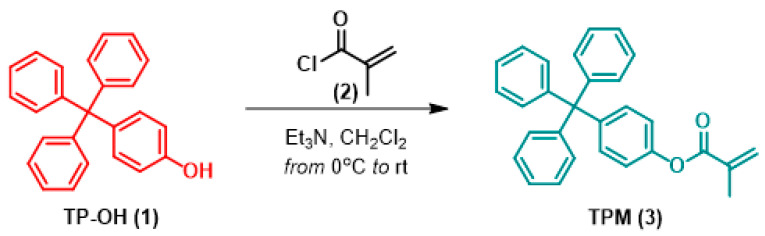
Reaction synthesis route for 4-TPM monomer. (1) 4-Tritylphenol, (2) Methacrylol chloride, (3) 4-TPM monomer.

The synthesis route for the 4-TPM monomer is shown in Figure 8. As illustrated, the synthesis of the target compound (3,4-TPM) was performed as outlined in the literature [27]. The 4-Tritylphenol (TP-OH, 1.0 g, 2.97 mmol) was dissolved in dry CH_2_Cl_2_ (50 mL). Triethylamine (Et_3_N) (1.5 equivalents, 0.60 mL) was then added to the solution. The reaction was kept at 0 °C while methacryloyl chloride (2, 1.5 equivalents, 0.43 mL) in CH_2_Cl_2_ (10 mL) was added dropwise via a dropping funnel over about 30 min.

The reaction mixture was stirred overnight while slowly being warmed to room temperature. After checking with TLC that the reaction had finished, the mixture was washed with 30 mL of aqueous sodium bicarbonate (NaHCO_3_), water and, finally, brine. The organic layer was then dried over sodium sulfate (Na_2_SO_4_), filtered and the solvent evaporated under vacuum. The crude product was further purified via flash column chromatography using a chloroform/hexane mixture (25%), yielding the target compound, 4-tritylphenyl methacrylate (4-TPM, 1.06 g, 88%) (see Figure S1). Finally, the synthesis of 4-TPM was achieved in accordance with the published literature [27] and the product was obtained as a pale white solid (see Figure S1). ^1^H-NMR (400 MHz, DMSO-d6): δ 7.29–7.32 (m, 6H, =CH), 7.19–7.23 (m, 4H, =CH), 7.09–7.16 (m, 9H, =CH), 6.25 (m, 1H, C=CH_2_), 5.88 (m, 1H, C=CH_2_), 1.98 (m, 3H, CH_3_); (see Figure S2) ^13^C-NMR (100 MHz, CDCl_3_): δ 18.67 (CH_3_), 64.79 (C=CH_2_), 121.59, 126.76, 128.47, 131.11, 132.19, 135.92, 144.56, 146.87, 146.93, 149.14, 165.83 (see Figure S3).

#### 3.3.2. In Situ Polymerization

Silanization is a preliminary process for preparing capillary monoliths. First, the empty 20 µm ID fused silica capillary was silanized in accordance with the published literature [28]. The polymerization mixture consisted of 14.09% 4-TPM (*wt*/*wt*), 25.18% TRIM (*wt*/*wt*), 10.92% cyclohexanol (*wt*/*wt*), 49.01% dodecanol (*wt*/*wt*) and 0.8% AIBN (*wt*/*wt*). These components were mixed in a 1.5 mL Eppendorf tube. The 4-TPM content was optimized in polymerization solutions (see Table 1). The polymerization mixture was homogenized in an ultrasonicator for 5 min and injected into the silanized fused silica column. Both column ends were tightly plugged. The column was then placed in a water bath at 65 °C for 20 h. The final monolith was washed with ACN:H_2_O (80:20, *v*/*v*) at a flow rate of 200 nL/min for 4 h before use.

### 3.4. Protein-Peptide and Cell Culture Preparations

The sample protein mixture was prepared by taking 100 μL aliquots from the stock solutions to obtain a final concentration of 0.020 mg/mL for each of the seven proteins. The proteome sample for the MCF-7 cell lines was prepared according to our previous published article [8].

## 4. Conclusions

This study presents the development and evaluation of a new 4-TPM-based monolithic nano-column with an inner diameter of 20 µm for ultra-sensitive bioanalytical separations by nano-LC. The monolith composition was systematically optimized with respect to the 4-TPM content and porogenic solvent, resulting in the TPM 6 formulation. This formulation provides favourable permeability, a high specific surface area and good mechanical stability. Chromatographic characterization using alkylbenzenes and polycyclic aromatic hydrocarbons confirmed that the stationary phase exhibits strong hydrophobicity, effective π–π interactions and electron-donating properties. The optimized TPM 6 column enabled the efficient separation of three model dipeptides and seven intact proteins under low nanoliter flow conditions, demonstrating its suitability for peptide and protein analysis. When applied to a tryptic digest of the MCF-7 cell line, the column delivered high peak capacity and enhanced peptide detection compared to a commercially available nano-LC column with the same inner diameter, highlighting its potential for trace and top-down proteomics. Overall, the 4-TPM monolithic nano-column is a robust, highly sensitive platform for nano-LC separations of complex biomolecular samples, offering a promising alternative to existing miniaturized stationary phases in advanced omics applications.

## Figures and Tables

**Table 1:** Composition of the polymerization solutions used for 4-TPM monolith preparation.

Column	Monomers (Molar Ratio)	Porogen (µL)	R^2 a^	Specific Surface Area (m^2^/g)	Permeability (×10^−14^)
	4-TPM: TRIM	Cyclohexanol: 1-Dodecanol			
TPM 1	1.0:2.1	350:150	-	-	-
TPM 2	1.0:2.1	300:200	-	-	-
TPM 3	1.0:2.1	250:250	-	-	-
TPM 4	1.0:2.1	200:300	-	-	too low flow
TPM 5	1.0:2.1	100:400	0.9994	141.4	4.34
TPM 6	1.2:2.1	100:400	0.9997	211.7	1.30
TPM 7	1.4:2.1	100:400	-	-	-
TPM 8	1.6:2.1	100:400	-	-	-

- no calculation, ^a^ linear relationship between flow rate and the resulting backpressure of relevant monolith.

## Data Availability

The original contributions presented in this study are included in the article/Supplementary Materials. Further inquiries can be directed to the corresponding author.

## Supplementary Materials

The following supporting information can be downloaded at: https://www.mdpi.com/article/10.3390/ijms27010224/s1.
