# Supplementary material for: Ultra-Sensitive Bioanalytical Separations Using a New 4-Tritylphenyl Methacrylate-Based Monolithic Nano-Column with an Inner Diameter of 20 µm for Nano-LC
Source: Int J Mol Sci. 2025 Dec 25;27(1):224. doi: 10.3390/ijms27010224 (PMC12785336; doi:10.3390/ijms27010224)
Supplement: Supplementary file 1 [file ijms-27-00224-s001.zip › ijms-4034399-supplementary.pdf]

# SUPPLEMENTARY MATERIAL FOR

## Ultra-sensitive bioanalytical separations using a new 4-tritylphenyl methacrylate-based monolithic nano-column with an inner diameter of 20 µm for nano-LC

**Cemil Aydoğan**

Food Analysis and Research Laboratory, Bingöl University, Bingöl, Türkiye
Department of Food Engineering, Bingöl University, Bingöl, Türkiye
Department of Chemistry, Bingöl University, Bingöl, Türkiye

*Correspondence should be addressed to the following author:

*Prof. Dr. Cemil Aydoğan*
*Dean of Engineering and Architecture Faculty*
*Bingöl Üniversity*
+904262160012/6112 *Bingöl / Türkiye*
Fax: +90 426 216 00 33
E-mail: caydogan@bingol.edu.tr

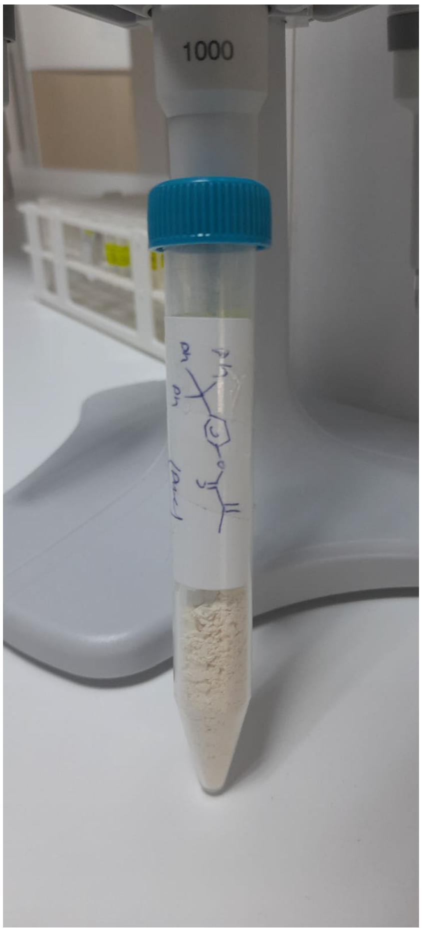

**Figure S1.** Synthesized 4-TPM monomer obtained as pale white solid

**4-TPM synthesize procedure:**

The 4-tritylphenol (TP-OH, 1, 1.0 gr, 2.97 mmol) was dissolved in dry $CH_2Cl_2$ (50 mL), and following triethylamine ($Et_3N$) (1.5 equiv., 0.60 mL) was added to the solution. While the reaction was kept at 0 ºC, the methacryloyl chloride (2, 1.5 equiv. 0.43 mL) in $CH_2Cl_2$ (10 mL) was added drop-wise for about 30 min. via dropping funnel. The reaction mixture was stirred overnight slowly warming to room temperature. The reaction progress and 4-TPM monomer synthesis were monitored by TLC on silica gel plates using an ethyl acetate/hexane (1:1) solvent system. Completion was confirmed by disappearance of the starting material spot and appearance of the product spot with the expected Rf value. After checking with TLC whether the reaction was finished or not, the reaction mixture was washed with 30 mL of aqueous sodium bicarbonate ($NaHCO_3$), water, and finally, brine. Following, the organic layer was dried over sodium sulfate ($Na_2SO_4$), filtered, and the solvent was evaporated in vacuo. The crude

product was further purified via flash column chromatography with $CH_2Cl_2$/hexane (25%), and the target 4-tritylphenyl methacrylate (TPM, 1.06 gr, 88%) was obtained.

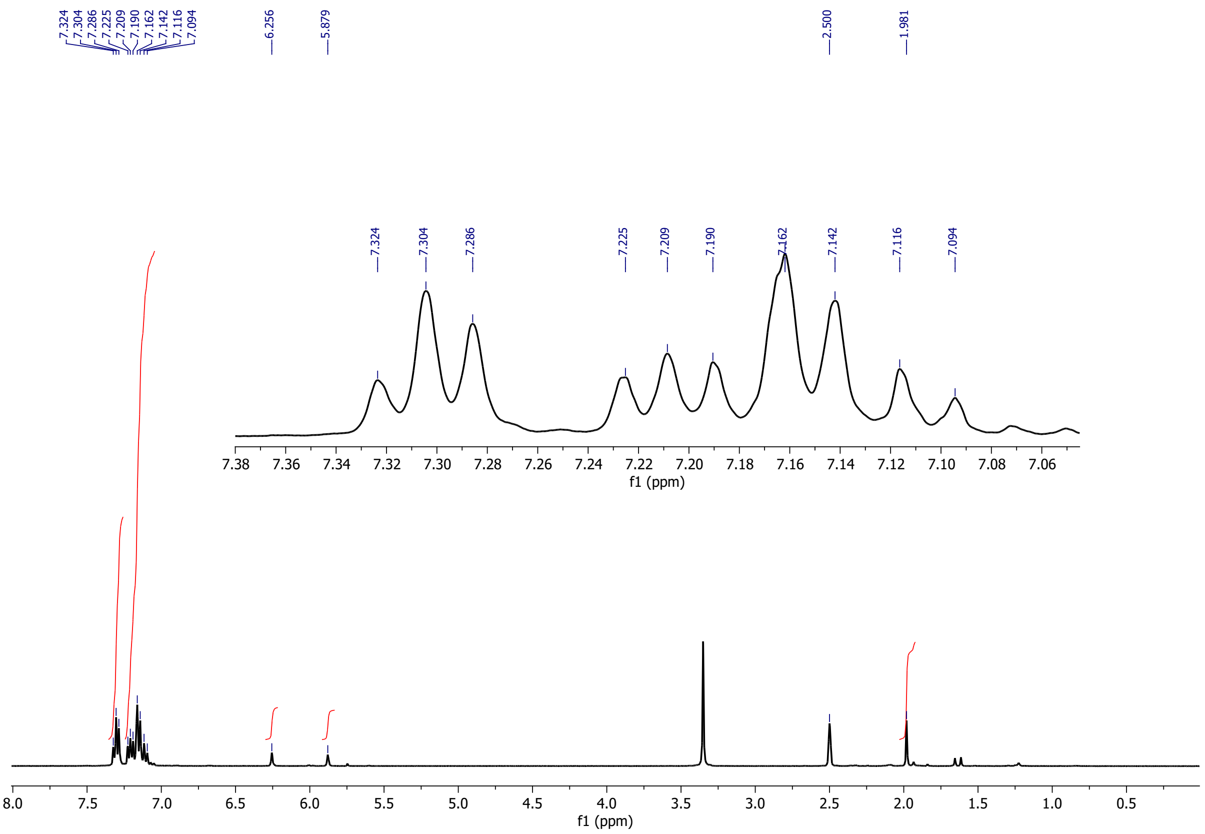

**Figure S2.**$^{1}$H-NMR (400 MHz) for 4-TPM monomer

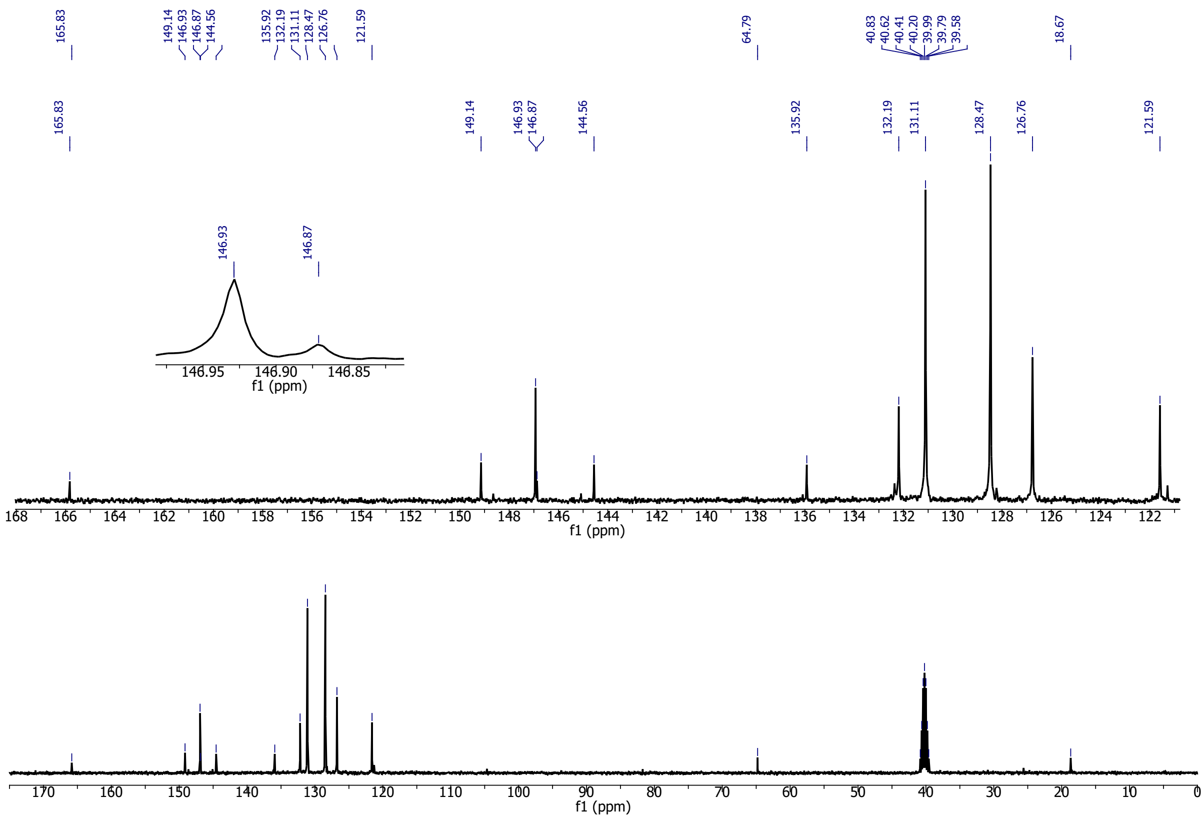


**Figure S3.** $^{13}$C-NMR (100 MHz) (DMSO-d6) for 4-TPM monomer.

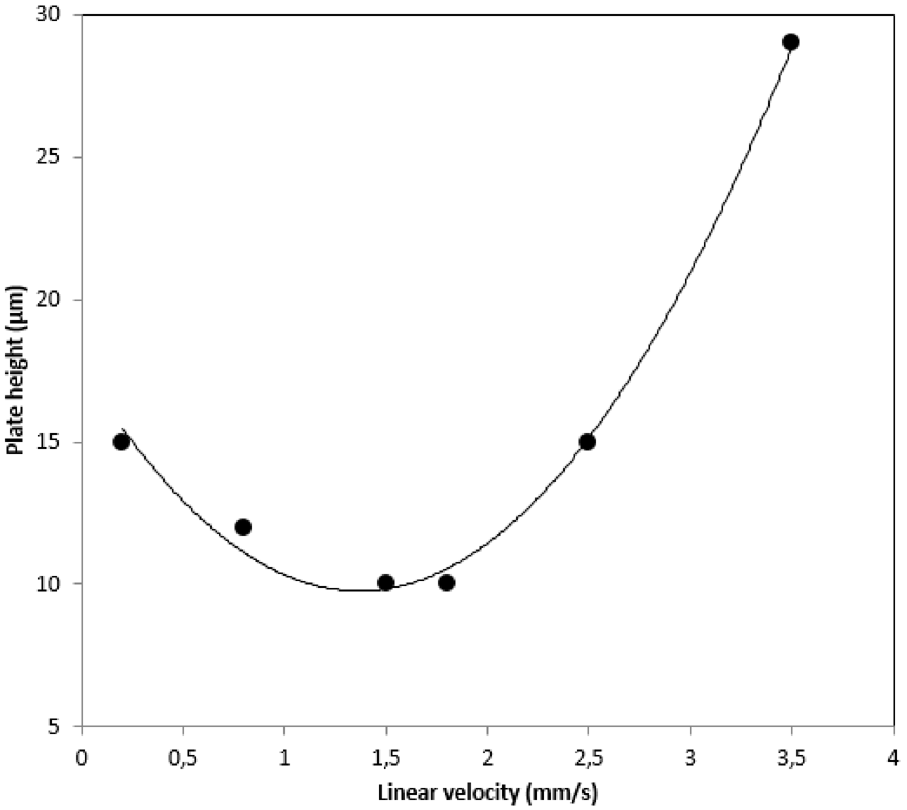


**Figure S4.** The linear velocity on the plate height of TPM 6 monolith using ACN $H_2O$ (80/20 % (v/v) for the separation of ABs derivatives.

Date: 03 Kasým 2025 Pazarte

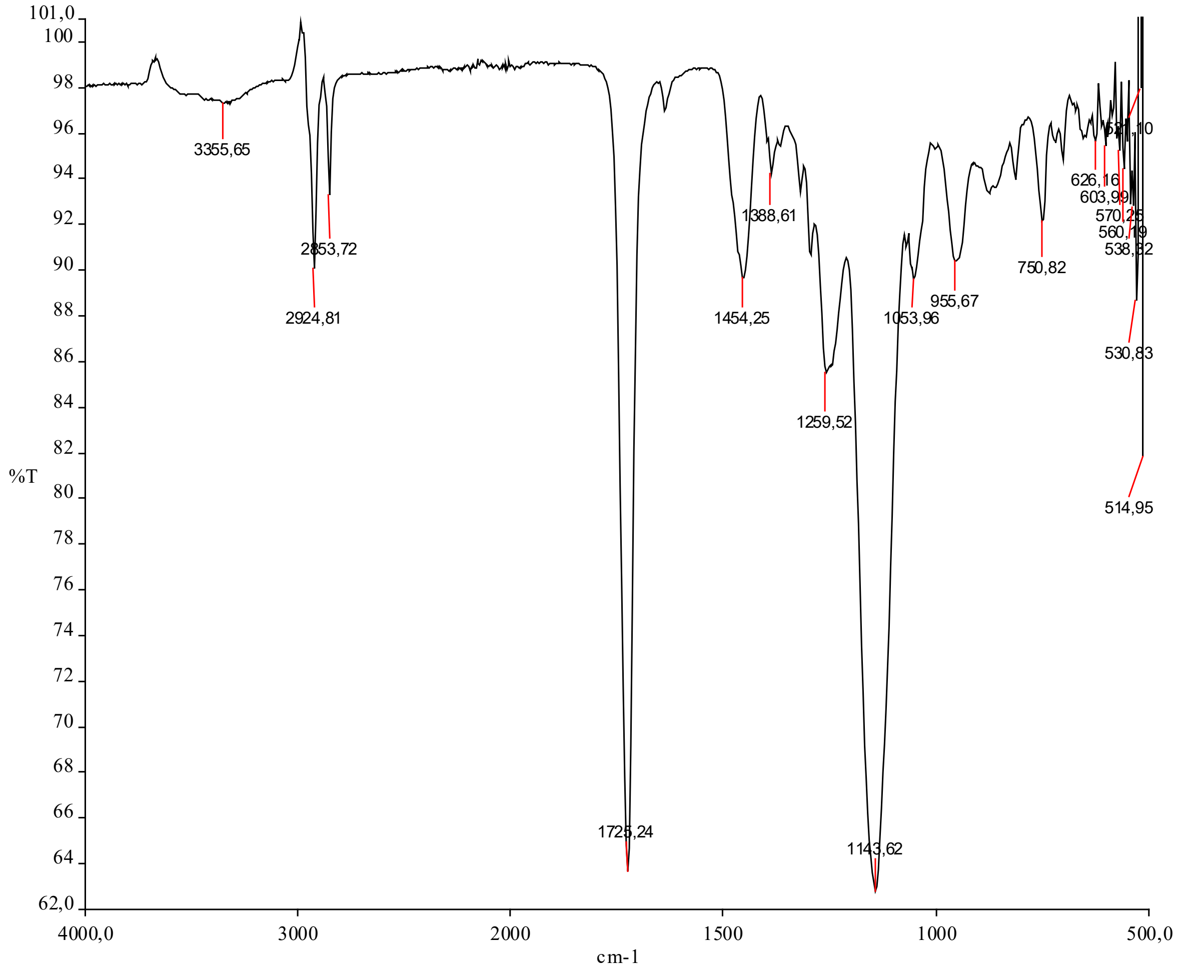


**Figure S5.** FT-IR spectra of the developed TPM-6 column

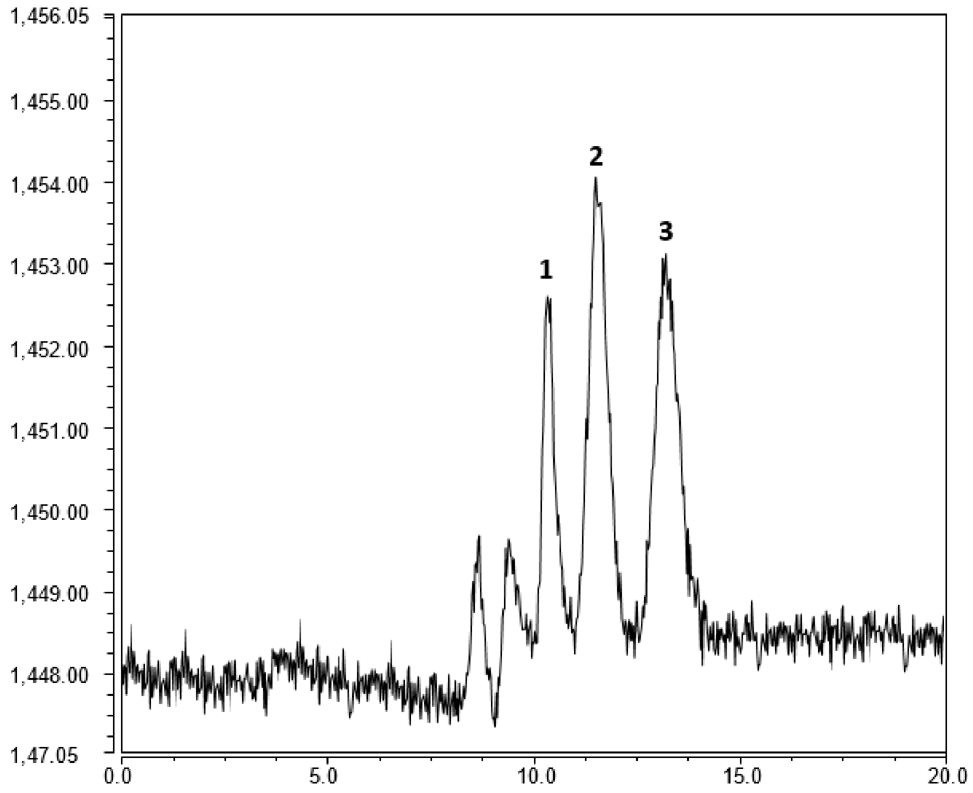


**Figure S6.** The chromatograms of peptide separation with the mobile phase (40/60 (v/v %) 50 mM phospahte buffer/ACN at pH 7.0) detection wavelegth; 214 nm, order of peaks; (1) L-carnosine (2) Ala-Tyr (3) Gly-Phe.
